# Occurrence of bioluminescent and nonbioluminescent species in the littoral earthworm genus *Pontodrilus*

**DOI:** 10.1038/s41598-021-87984-4

**Published:** 2021-04-16

**Authors:** Teerapong Seesamut, Daichi Yano, José Paitio, Ikuhiko Kin, Somsak Panha, Yuichi Oba

**Affiliations:** 1 Department of Environmental Biology, Chubu University, Kasugai, 487-8501 Japan; 2 Animal Systematics Research Unit, Department of Biology, Faculty of Science, Chulalongkorn University, Bangkok, 10330 Thailand; 3 Academy of Science, The Royal Society of Thailand, Bangkok, 10300 Thailand

**Keywords:** Ecology, Biocatalysis

## Abstract

*Pontodrilus litoralis* is a cosmopolitan littoral earthworm known to exhibit bioluminescence. Recently, a congeneric species, *Pontodrilus longissimus*, from Thailand was described. These species are sympatric, but their burrowing depths on Thai beaches are different. In this study, we examined the in vivo and in vitro bioluminescent properties of *P. longissimus* and *P. litoralis*. Mechanical stimulation induced in vivo luminescence in *P. litoralis*, as reported previously, but not in *P. longissimus*. In vitro cross-reaction tests between these species revealed the absence of luciferin and luciferase activities in *P. longissimus*. The coelomic fluid of *P. litoralis* had strong fluorescence that matched the spectral maximum of its bioluminescence, but the same result was not observed for *P. longissimus*. These results suggest that *P. litoralis* has luminescence abilities due to the creation of bioluminescent components (i.e., luciferin, luciferase, and light emitters). The presence of both luminous and nonluminous species in a single genus is likely widespread, but only a few examples have been confirmed. Our findings provide insight into the possible functions of bioluminescence in earthworms, such as avoiding predation by littoral earwigs.

## Introduction

The earthworm genus *Pontodrilus* Perrier, 1874, displays various unique characteristics. The littoral earthworm *P. litoralis* (Grube, 1855) is distributed on the tropical and subtropical coasts of the Atlantic, Indian and Pacific Oceans^1–3^ and is known to be bioluminescent^4–7^. The luminescent system of *P. litoralis* has been shown to be a luciferin-luciferase type reaction triggered by hydrogen peroxide, with a fluorescence compound acting as a light emitter^7^, although the chemical structure of the luciferin remains uncertain and the luciferase gene has not been determined. Recently, the littoral earthworm *Pontodrilus longissimus* Seesamut & Panha, 2018 was identified in coastal areas in Thailand and peninsular Malaysia^8^ and determined to be a separate species based on morphological differences from other congeners in the size of the body, the number of segments and the diverticulum.

In the present study, the bioluminescence and fluorescence of *P. litoralis and P. longissimus* were examined in vivo and in vitro, and the results suggested that *P. longissimus* lacks luminescence ability despite its genetically close relationship to *P. litoralis*. Based on these findings, we discuss the biological function of earthworm bioluminescence and a convenient parataxonomic method for *Pontodrilus* species.

## Results

### In vivo and in vitro bioluminescence

**Figure 1:**
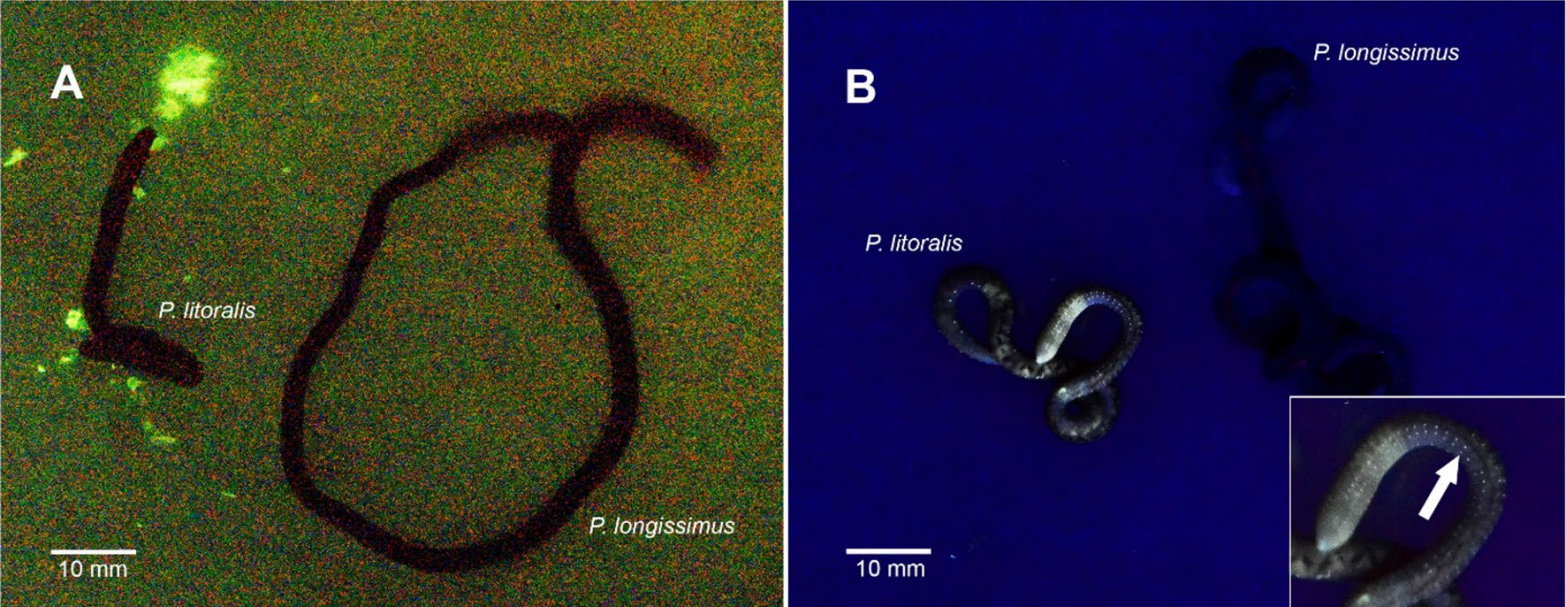
(**A**) Bioluminescence of littoral earthworms after mechanical stimulation. (**B**) Fluorescence observed under a handheld longwave UV lamp (365 nm). Inset: magnified image of *P. litoralis*. The arrow indicates a row of setae.

After the live specimens of both *Pontodrilus* species were stimulated by electricity or rough handling, *P. litoralis* exuded a green luminescent fluid, whereas the fluid exuded from *P. longissimus* was not luminescent (Fig. 1A). Under a handheld longwave UV lamp (365 nm), almost the entire body of *P. litoralis* emitted strong yellow fluorescence, which was most conspicuous in the rows of setae, whereas *P. longissimus* did not emit fluorescence under the same conditions (Fig. 1B).

**Figure 2:**
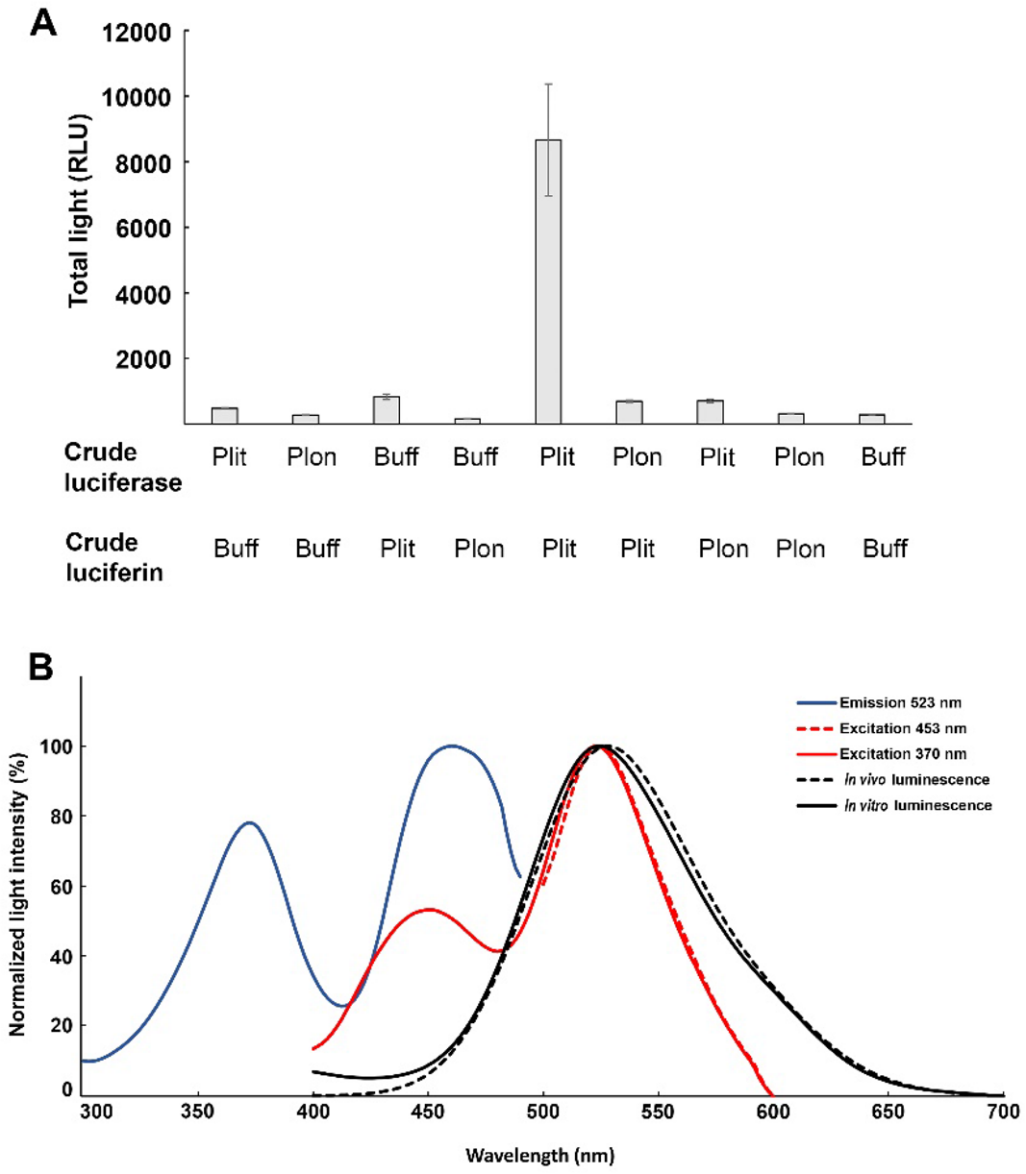
(**A**) Cross-reaction between crude luciferase and luciferin of the littoral earthworm genus *Pontodrilus*. Plit represents *P. litoralis*, Plon represents *P. longissimus*, and Buff represents the buffer (50 mM Tris–HCl, pH 7.2). Data are shown as the mean ± standard deviation (*n* = 3). (**B**) Fluorescence spectra of crude coelomic fluid extracted from *P. litoralis*. An excitation spectrum (solid blue line) with emission at 523 nm, an emission spectrum (dashed red line) with excitation at 453 nm, and an emission spectrum (solid red line) with excitation at 370 nm were obtained. The dashed black line shows the in vivo luminescence spectrum of live *P. litoralis*. The solid black line shows the in vitro luminescence spectrum of crude coelomic fluid extracted from *P. litoralis*, which contained 500 µl 50 mM Tris–HCl at pH 7.2 and 50 µl 0.3% hydrogen peroxide. The data shown are the means of five measurements.

The cross-reactivities of crude luciferase and crude luciferin in *P. litoralis* and *P. longissimus* were examined (Fig. 2A). To ensure the validity of the results, we used a concentration of *P. longissimus* extract that was fivefold higher than the concentration of *P. litoralis* extract. The results showed that significant luminescence was detected only when mixing the luciferin extract from *P. litoralis* with the luciferase extract from *P. litoralis*. On the other hand, both luciferin and luciferase activities in the extracts of *P. longissimus* were negative (the levels of both activities were almost the same as those in a negative control).

### Fluorescence and luminescence spectra of luminous *P. litoralis*

Fluorescence spectra were measured using a crude coelomic fluid extracted from *P. litoralis* (Fig. 2B). The peaks of the excitation spectra were 370 and 453 nm, whereas the emission peaks were 450 and 523 nm, indicating the presence of at least two fluorescent compounds in *P. litoralis*; however, the possibility of the presence of a single dual-chromophore molecule, such as a genetically designed biosensor, cannot be excluded. The luminescence spectra of *P. litoralis* had maximum wavelengths of 528 nm in vivo and 524 nm in vitro (Fig. 2B). Wampler and Jamieson showed that the spectral maximum of bioluminescence (540 nm) in Bermudan *P. bermudensis* (which is now considered synonymous with *P. litoralis*^2^) matched the fluorescence maximum of the coelomic fluid and suggested that the fluorescent substance is a light emitter^7^. Although the maximum spectral values in our study were different from those in their results, probably due to genetic differences in the specimens examined or differences in the spectrophotometers used, our results also showed a spectral match between fluorescence and bioluminescence in vitro. The small red shift in the in vivo spectrum might have been due to a reflection effect from the reddish earthworm body.

### Comparison of the coelomic fluid cells and protein bands between the two littoral earthworm species

**Figure 3:**
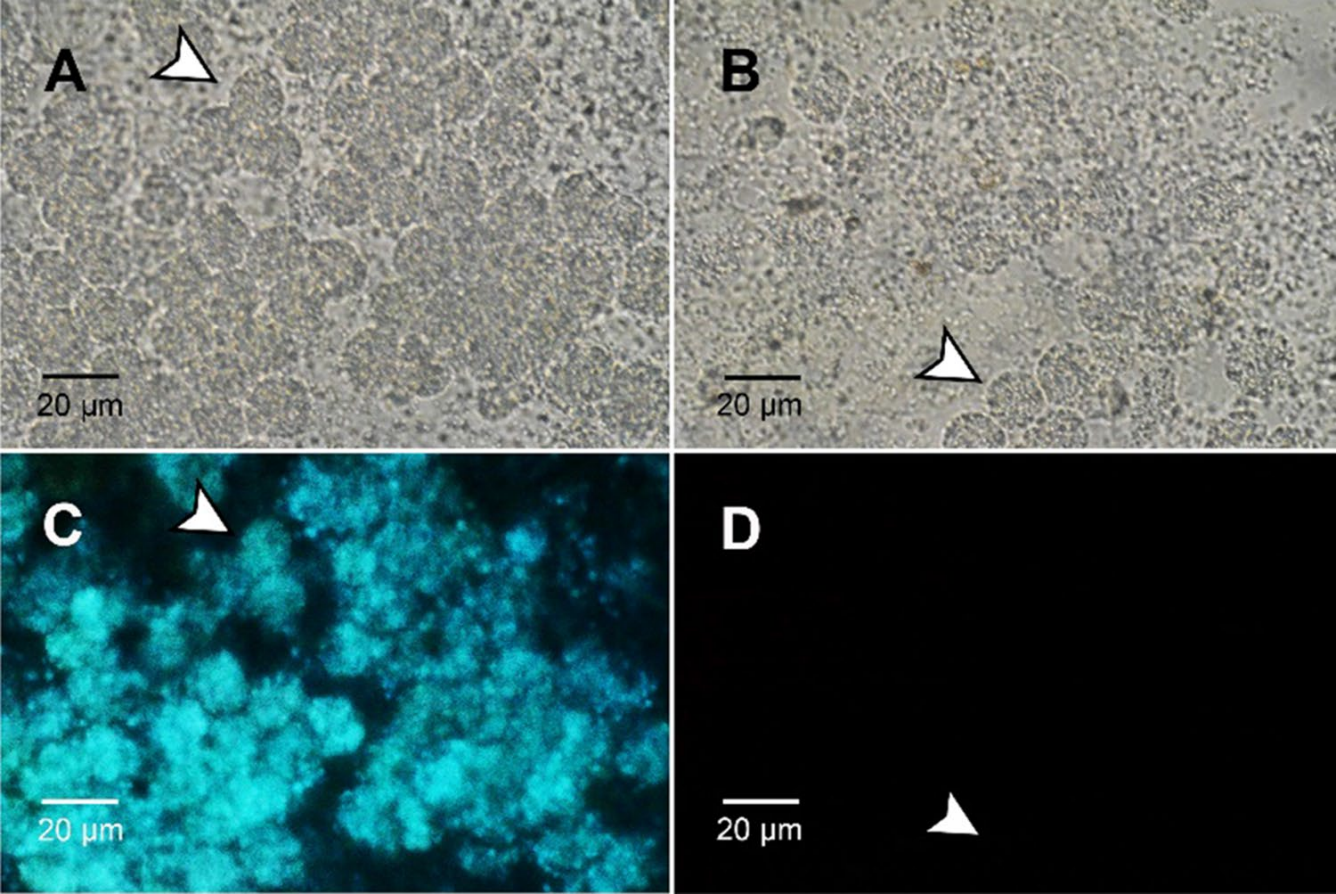
(**A**) Bright field image of *P. litoralis* coelomic cells (arrowheads). (**B**) Bright field image of *P. longissimus* coelomic cells. (**C**) *P. litoralis* coelomic cells under UV irradiation at 380 nm. (**D**) *P. longissimus* coelomic cells under UV irradiation at 380 nm. The photography conditions in (**C**) were the same as those in (**D**), but no fluorescence was observed in the latter image.

The coelomic cells of the littoral earthworm species were observed under a fluorescence microscope (Fig. 3). The results showed that the coelomic cells of *P. litoralis* emitted fluorescence, while those of *P. longissimus* did not. The diameter of the coelomic cells was approximately 15 µm, and numerous small fluorescent particles were detected in the coelomic cells of *P. litoralis*. SDS-PAGE of the coelomic fluids showed different protein constitutions between the two species (Supplementary Fig. S1).

## Discussion

In this study, we confirmed that *P. longissimus* is nonbioluminescent, despite its close relationship to the luminous species *P. litoralis* (Supplementary Fig. S2)^8^. The presence of both luminous and nonluminous species in a single genus is likely widespread, but only a few examples have been confirmed; for example, the genera *Vibrio* and *Photobacterium* (marine bacteria)^9^, *Epigonus* (deep-sea fishes)^10^, *Mycena* (bonnet mushrooms)^11^ and *Eisenia* (terrestrial earthworms)^12^ have been reported to contain both luminous and nonluminous species. *P. litoralis* and *P. longissimus* can easily be collected at the same beach^8^ and reared in a laboratory; thus, they are suitable for studying the ecology and evolution of bioluminescence.

In vitro luciferin-luciferase cross-reaction tests of *P. longissimus* and *P. litoralis* confirmed that the luminescence ability of *P. litoralis* is due to the presence of multiple bioluminescent components in coelomic fluid, i.e., luciferin, luciferase and the light emitter. Cross-reaction tests have previously indicated that luminous earthworms in the genera *Pontodrilus* (Megascolecidae), *Microscolex* and *Diplocardia* (Acanthodrilidae) share the same basic bioluminescence mechanisms^5,7,13,14^, despite their distant relationships to each other^15,16^. It is expected that the ancestral state of *Pontodrilus* is nonbioluminescent because the nearest extant relatives of *Pontodrilus* belong to the genus *Plutellus* Perrier, 1873, and all members of this group are nonbioluminescent^6,17^. These findings suggested that *P. litoralis* secondarily acquired bioluminescent properties through parallel evolution, similar to the case of bioluminescence in lampyrid and elaterid beetles^18^. We detected a clear difference in the protein composition of the secreted fluid between *P. litoralis* and *P. longissimus* (Supplementary Fig. S1). Luciferase and other bioluminescent components of luminous earthworms were not identified, and further comparative analyses of the protein bands, which appear only in the secreted fluid of luminous species, will be useful to understand the mechanisms of bioluminescence and its parallel evolution.

**Figure 4:**
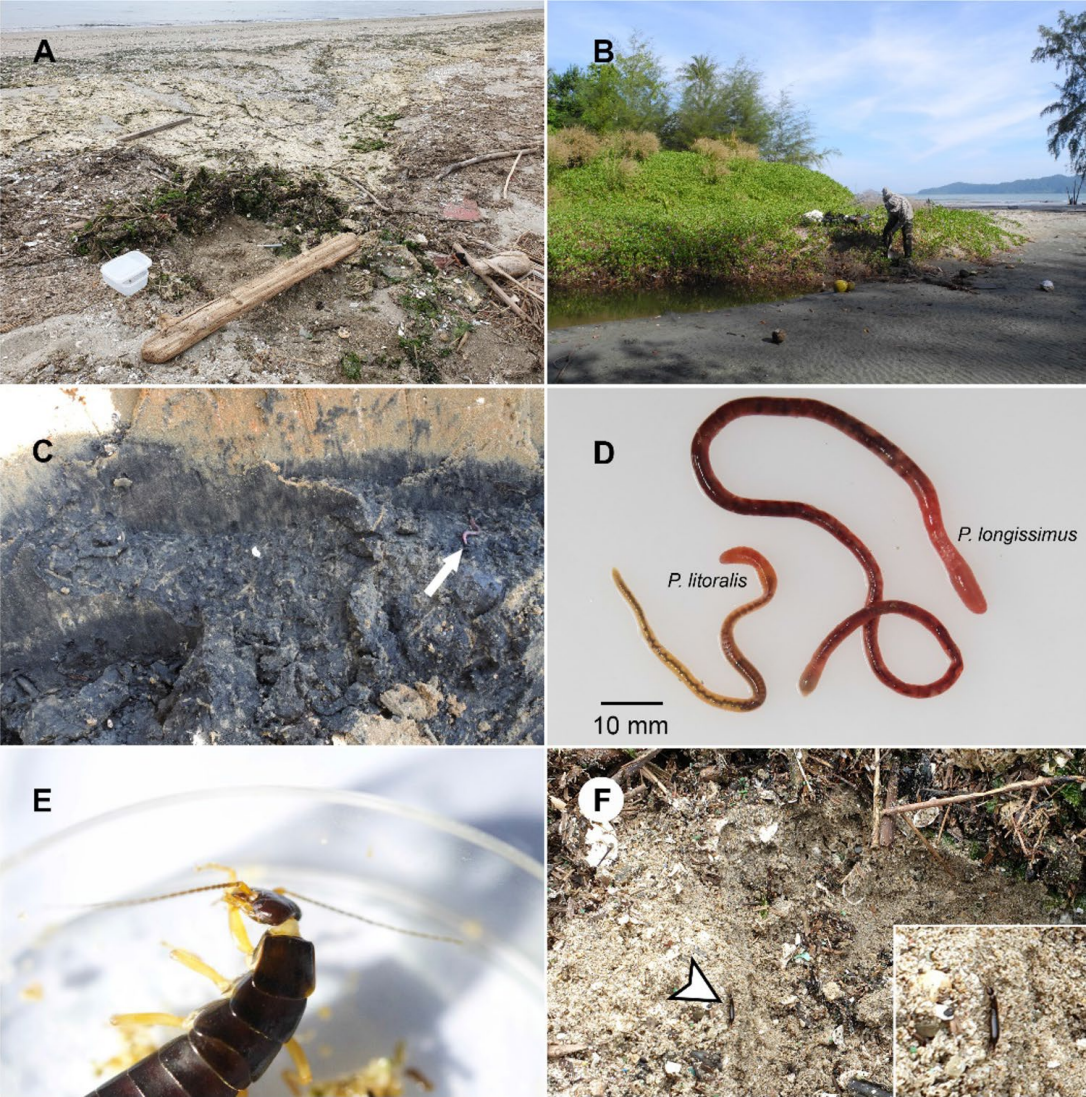
(**A**) The microhabitat of *Pontodrilus litoralis* from Aichi Prefecture, Japan. (**B**) The microhabitat of *P. longissimus* in Ranong, Thailand; sympatric *Pontodrilus* specimens were collected from this location^8^. (**C**) *P. longissimus* was found at a depth of 10–30 cm in muddy sand; the earthworm is shown by an arrow. (**D**) Bright field image of the *Pontodrilus* species included in this study. (**E**) An earwig (*Anisolabis maritima*) (a potential *Pontodrilus* predator) grooming its forelegs after attacking *P. litoralis*. (**F**) *A. maritima* (arrowhead) was found in the same microhabitat as *P. litoralis* in Aichi Prefecture, Japan.

In Thailand*, P. longissimus* was found sympatrically with *P. litoralis* at the beaches along the coast, but the microhabitats of the two congeners are different; *P. litoralis* was collected on the beach surface (under trash or leaf litter on sandy beaches), whereas *P. longissimus* was found at a greater depth than *P. litoralis*, i.e., a depth of more than 10 cm, where trash and leaves are scarce^8^ (Fig. 4A–D). It has been hypothesized that the biological function of bioluminescence in Annelida, including *P. litoralis*, is to stun or divert attention as an anti-predator defense^19–25^, but experiments and observations of the prey are limited. Sivinski & Forrest^25^ reported that the luminescence of *Microscolex phosphoreus* deterred predation by the mole cricket *Scapteriscus acletus* under laboratory conditions, although the specimen was ultimately consumed. A British television program^26^ presented by David Attenborough showed that the French luminous earthworm *Avelona ligra* glowed when attacked by the carabid beetle, but the beetle consumed the luminescent worm without any hesitation. We suggest that the absence of bioluminescence in *P. longissimus* may be associated with its presence in habitats with low predation pressure, whereas *P. litoralis* acquired a bioluminescent property during evolution that enabled it live on the surface of the beach, which is rich in nutrition and food sources^3,27^ as well as potential predators.

Indeed, while we observed some burrowing bivalves, no potential predators were observed in the deep sand inhabited by *P. longissimus*. In contrast, various carnivorous invertebrates, such as earwigs, rove beetles and carabid beetles, were observed on the surface of beaches in Thailand and Japan, where *P. litoralis* live (Seesamut pers. obs.). We therefore performed a feeding experiment using maritime earwigs sympatrically distributed in a *P. litoralis* habitat. The maritime earwig *Anisolabis maritima* (Dermaptera, Anisolabididae) is a cosmopolitan species that can be found in Japan. It has well-developed compound eyes (Fig. 4E) and is considered a carnivorous animal that forages for prey at night^28, 29^. *A. maritima* (body length ≤ 30 mm) was the predominant predator at the beach where *P. litoralis* was collected (Fig. 4F). Some rove beetles (Coleoptera, Staphylinidae) were found in the same habitat, but they seemed to be too small (< 10 mm) to consume *P. litoralis,* and during our laboratory observations, the rove beetle did not attack the worm. Thus, we think *A. maritima* is a major potential predator of *P. litoralis* at the beaches in Japan. Live *P. litoralis* and *A. maritima* were collected from the same beach on the same day, and we observed the predation behavior by the latter in a laboratory within a dark cage with beach sand spread on the bottom. Our observation of the predation of *P. litoralis* by earwigs (Supplementary Video 1) may provide important insight into the function of bioluminescence in *P. litoralis*. The earwigs immediately began aggressively attacking the worm with their mandibles and abdominal cerci, a pair of scissor-like pincers; the worm secreted luminescent mucus from its wounds (Supplementary Video 1), and it appeared that the retention of the glue-like luminescent mucus on the mouth and forelegs of the earwigs was unpleasant to them, since they attempted to remove the mucus by frequent grooming (Fig. 4E, Supplementary Video 2). Indeed, after aggressive attacks, the earwigs ultimately abandoned their attempts to consume the worm, and thus, the worm survived. To the best of our knowledge, this is the first observation of earthworm bioluminescence induced by predation under almost natural conditions. Based on these observations, we hypothesized that the luminous glue-like mucus of *P. litoralis* may function to deter and/or divert predation and that luminescence might even enhance the avoidance learning of the predator. Notably, the amount of mucus exuded following the same mechanical stimulation seemed to be much higher in *P. litoralis* than in *P. longissimus*. Nevertheless, we suppose that the global distribution of *P. litoralis* is a consequence of its adaptation to the beach surface (i.e., luminescence), which provides opportunities for dispersal by current, whereas *P. longissimus* is endemic to the coasts of the Thai-Malay peninsula^8,30^ due to its inhabitation of deeper sand.

Based on microscopic observations, we confirmed that both species secrete coelomic cells following stimulation, but neither bioluminescence nor fluorescence was observed in *P. longissimus*. The presence and absence of fluorescence in a single genus of earthworm was also reported in the terrestrial genus *Eisenia*, and it has been suggested that the difference in fluorescent characteristics could be used as a “fluorescence fingerprint” to delineate these closely related species^31^. Therefore, the fluorescence fingerprint method is also applicable to *Pontodrilus*.

Littoral zones have rich species diversity of both macro- and microorganisms^32,33^. They comprise a front of human pressure in marine ecology and are one of the most important zones for conservation^34,35^; therefore, there is a need to understand littoral fauna. Earthworms typically have strong effects on soil ecosystems^36–38^. *Pontodrilus* is a major “ecosystem engineer”^38^ that inhabits littoral habitats. Thus, the identification of *P. litoralis* and *P. longissimus* is significant to the assessment of the littoral environment. These species are actually distinguishable by the internal morphology of the spermathecal diverticulum, but special skills and equipment are necessary for morphological analyses. In this study, we identified differences in the bioluminescent and fluorescent characteristics of *P. litoralis* and *P. longissimus* and demonstrated that the analysis of these differences provides an easy in situ methodology to identify these earthworms in marine ecological studies and for the conservation of littoral zones in Southeast Asia.

## Methods

### Specimens and species identification

The littoral earthworm *P. litoralis* was collected at a sandy region of one of the following beaches^3,8^: Wonnapa Beach, Amphoe Mueang Chon Buri, Chonburi, Thailand (13°15′55.6"N 100°55′29.3"E); Kowa Beach, Chita, Aichi Prefecture, Japan (34°46′23.3"N 136°54′52.7"E); and Kira Waikiki Beach, Nishio, Aichi Prefecture, Japan (34°46′55.2"N 137°05′48.3"E). *P. longissimus* was collected from Tambon Muang Klang, Amphoe Kaper, Ranong, Thailand (9°37′26.7"N 98°28′08.6"E). The earthworms were maintained in native sand in plastic containers sprayed with artificial seawater to keep the sand moist. Species identification was performed based on morphological characteristics by Seesamut et al.^8^. In vivo bioluminescence was photographed in darkness with a Nikon D5500 digital camera (Nikon, Japan). *Pontodrilus* were stimulated by electricity or rough handling to induce bioluminescence, and in vivo fluorescence was photographed under a handheld UV lamp (365 nm) without mechanical stimulation.

### Extraction of the luminescent substance

To prepare crude *Pontodrilus* luciferase and luciferin, live earthworms were rinsed with distilled water and transferred for 24 h to Petri dishes with wet tissue paper moistened with artificial seawater to avoid contamination by their stomach contents when extracting coelomic fluid. All experiments were carried out on ice except for the measurements of light intensity and spectra. Coelomic fluid was extracted as follows: twenty live worms of each species (2.72 g wet weight of *P. litoralis* and 7.4 g wet weight of *P. longissimus*) were put on a mortar and stimulated with a pestle to induce exudation of coelomic fluid, and then 10 ml of 50 mM Tris–HCl at pH 7.2 was added. After removing the specimens, the solution was centrifuged at 15,000 × g for 15 min at 4 °C in a TOMY MX-100 high speed refrigerated microcentrifuge (Tomy Seiko, Japan), and the supernatants were collected as the crude extracts. The crude luciferin and luciferase fractions were prepared based on the method described by Bellisario et al.^39^. In brief, the crude extracts were filtered using a 10 K centrifugal filter device (Merck, Germany); the first flow through was used as a crude luciferin extract, and the retentates on the membranes were collected as crude luciferase extract.

### Cross-reaction experiment and spectral measurement

The total protein concentrations of the crude luciferase extracts measured using a protein assay kit (Bio-Rad, USA) were 19.56 µg/ml in *P. litoralis* and 102.78 µg/ml in *P. longissimus*. The luminescent activity was monitored using a luminometer (Centro LB960, Berthold). Ten microliters of crude luciferase was mixed with 40 µl of crude luciferin, and 10 µl of 0.3% hydrogen peroxide was injected to initiate the luminescence reaction. The luminescence was recorded in relative light units (RLUs) for a total of 120 s after hydrogen peroxide injection. Three replicate measurements were performed.

### Spectral measurement

Luminescence and fluorescence spectra were recorded with a spectrofluorometer (JASCO, FP-777 W). For the luminescence spectra measurements, the excitation light source was shut off. The data were smoothed using a binomial method, and the spectral response was not corrected. An in vivo luminescence spectrum was obtained using a single living specimen put into a quartz cuvette immediately after stimulation by rough handling. To obtain an in vitro luminescence spectrum, 100 µl of crude luciferase and 300 µl of luciferin were mixed with 400 ml of 50 mM Tris–HCl at pH 7.2 and 40 µl of 0.3% hydrogen peroxide, and luminescence was immediately measured. Fluorescence spectra of coelomic fluid from *P. litoralis* were obtained using crude extract suspended in 500 µl of 50 mM Tris–HCl at pH 7.2. The bandwidths used for emission and excitation were 5 nm.

### Coelomic cell photography and SDS-PAGE

Coelomic cells of *Pontodrilus* were isolated by stimulating earthworms on microscope slides, observed under a fluorescence microscope (Nikon Eclipse E600, Japan) with a 60 × objective lens (Nikon CFI Plan Fluor Series, Japan), and photographed using an attached digital camera (Nikon D5500, Japan). The fluorescence excitation was performed at 380 nm.

The crude coelomic extract of both species was run on a 15% SDS-PAGE gel using a 1D Gel Electrophoresis Mini Gel, AE-6530mPAGE (ATTO), followed by silver staining (Silver Stain MS Kit, FUJIFILM Wako Pure Chemical Corporation).

### Video recording

Video recording of the live specimens was performed using a Nikon D500 and Micro NIKKOR 60 mm lens (Nikon) under red light (LED Lenser T^2^QC) with the following settings: ISO, 64000; f/2.8; and exposure, 1/60 s.

## Supplementary Information

Supplementary Information 1.

Supplementary Video 1.

Supplementary Video 2.
