# Supplementary material for: Occurrence of bioluminescent and nonbioluminescent species in the littoral earthworm genus Pontodrilus
Source: Sci Rep. 2021 Apr 16;11:8407. doi: 10.1038/s41598-021-87984-4 (PMC8052329; doi:10.1038/s41598-021-87984-4)
Supplement: Supplementary file 1 — Supplementary Information 1. [file 41598_2021_87984_MOESM1_ESM.docx]

Supplementary Materials

**Occurrence of bioluminescent and nonbioluminescent species in the littoral earthworm genus *Pontodrilus***

Teerapong Seesamut^1^, Daichi Yano^1^, José Paitio^1^, Ikuhiko Kin^1^, Somsak Panha^2, 3^, Yuichi Oba^1*^

^1^ Department of Environmental Biology, Chubu University, Kasugai 487-8501, Japan

^2^ Animal Systematics Research Unit, Department of Biology, Faculty of Science, Chulalongkorn University, Bangkok 10330, Thailand

^3^ Academy of Science, The Royal Society of Thailand, Bangkok 10300, Thailand

^*^Corresponding Author: Yuichi Oba

Email address: yoba@isc.chubu.ac.jp

**Supplementary Materials legends**

**Supplementary Figure S1.** Full-size gel images of SDS-PAGE of the crude coelomic extract of *Pontodrilus litoralis* (bioluminescent), *P. longissimus* (non-bioluminescent) and *Microscolex phosphoreus* (bioluminescent). Detection by silver staining. Molecular weight marker sizes in kDa are provided on the left. 10 and 2 indicate loading volume (µl). Note, a comparison of the band patterns between two luminous earthworms, *P. litoralis* and *M. phosphoreus* which share the same bioluminescence mechanism, will also be useful to determine the bioluminescence components.

**Supplementary Figure S2.** Maximum likelihood phylogenetic tree based on *COI* sequence of *Pontodrilus litoralis* and *longissimus* with megascolecid genera *Plutellus*, *Metaphire* and *Amynthas* available from GenBank. *COI* sequences were aligned using the ClustalW algorithm in MEGA X. ML analysis in RAxML v. 8, where 1000 bootstraps were used to estimate the node reliability to obtain bootstrap support values. Numbers around nodes are bootstrap values. Tree was rooted with *Diplocardia* (Acanthodrilidae) as outgroup. As shown in this tree, the genetic distance between *P. litoralis* and *longissimus* are closely related when it is compared with the distances among the species of *Metaphire*.

**Supplementary Video 1.** Observation of the predation of littoral earthworm *Pontodrilus litoralis* by the littoral earwig *Anisolabis maritima*.

**Supplementary Video 2.** *Anisolabis maritima* struggled to remove the gluey luminescent mucus by frequent grooming.


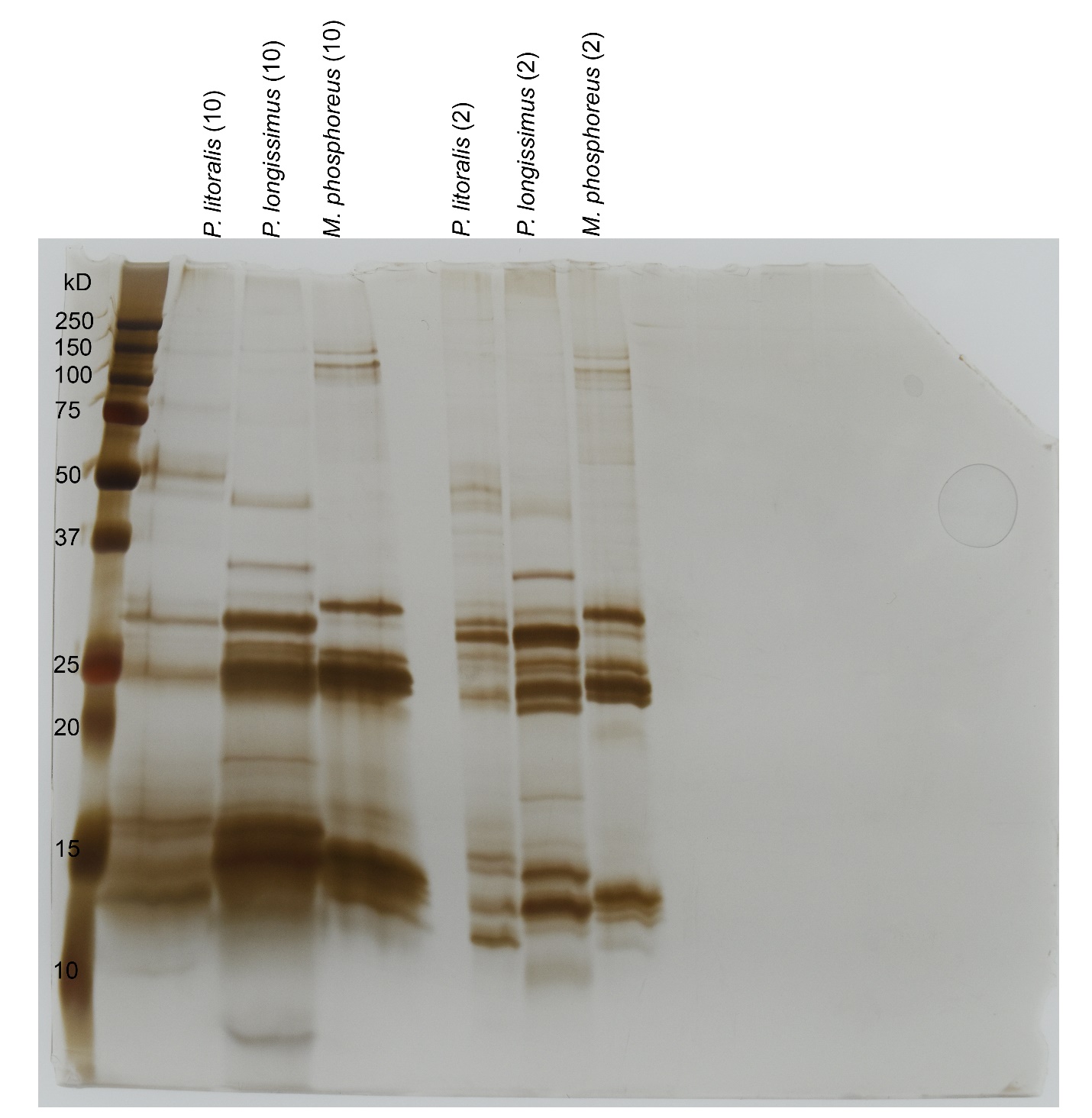


**Supplementary Figure S1.**


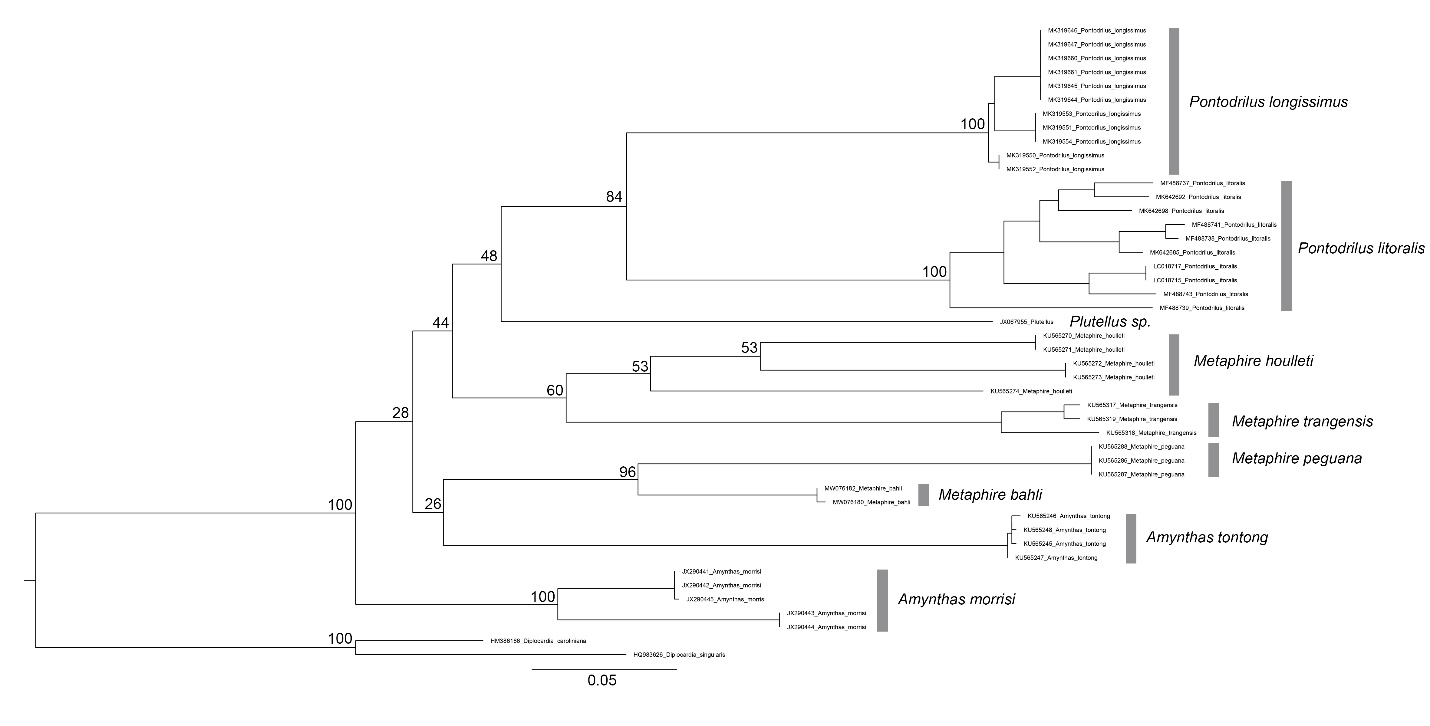


**Supplementary Figure S2.**
